# Predominantly Fibrous Malignant Mesothelioma in a Cat

**DOI:** 10.4061/2010/396794

**Published:** 2010-06-15

**Authors:** Alexander Th. A. Weiss, Afonso B. da Costa, Robert Klopfleisch

**Affiliations:** Department of Veterinary Pathology, Freie Universitaet Berlin, Robert-von-Ostertag-Str. 15, 14163 Berlin, Germany

## Abstract

Malignant mesotheliomas are rare tumours in domestic cats. They occur within the abdominal or thoracic cavity and are regularly associated with pleural or peritoneal effusions. The histopathological diagnosis can be quite challenging, as these neoplasms may resemble other epithelial or mesenchymal neoplasms. However, differentiation can be achieved by immunohistochemistry in most cases. Here we describe the rare case of a malignant mesothelioma of the fibrous subtype in the thoracic cavity of a cat and discuss differential diagnoses and treatment options for this tumor type.

## 1. Introduction

Spontaneous mesotheliomas are rare predominantly malignant neoplasms in domestic cats [1, 2]. They occur more frequently in young cattle and adult dogs and horses [3]. Mesotheliomas are of mesodermal origin and arise from the serosal surfaces of the pleura, peritoneum, and pericardium, as well as occasionally from the tunica vaginalis testis. Three main histological types are described in domestic animals: the epithelioid, the fibrous (spindle cell), and the biphasic (or mixed) type [3]. The epithelioid type is the most common in cats and resembles epithelial neoplasms. In contrast, the fibrous and the biphasic types occur less often and resemble other spindle cell tumours like fibrosarcomas. However, all three basic histological subtypes of mesothliomas occur as benign and malignant variants and prognosis is poor for all malignant types. 

Major differential diagnoses for mesothelioma in the cat are mediastinal lymphoma and fibrosarcoma. In human medicine the clinically benign reactive fibrosis is also a important differential diagnosis [1, 4]. This report describes a case of predominantly fibrous mesothelioma in a domestic short hair cat and discusses clinical features, diagnostical approaches, and differential diagnoses.

## 2. Case Description

An approximately 15 years old female domestic short hair cat was submitted for necropsy to our department. The animal had died spontaneously after several days of dyspnoea. Unfortunately, clinical diagnostic procedures were hindered by disobliging behavior of the cat.

**Figure 1:**
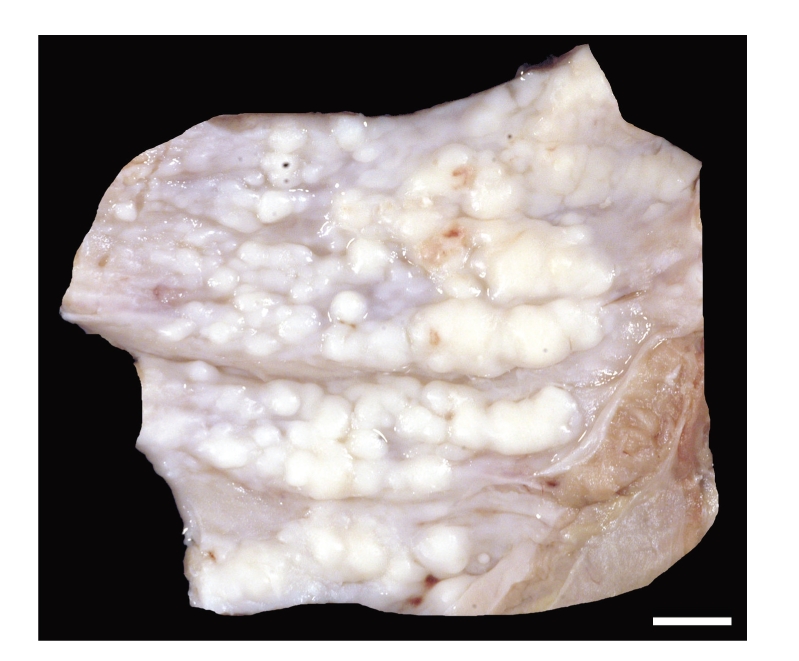
Thoracic wall, the pleural surface is covered by firm whitish nodules. Bar *≈* 1.5 cm.

**Figure 2:**
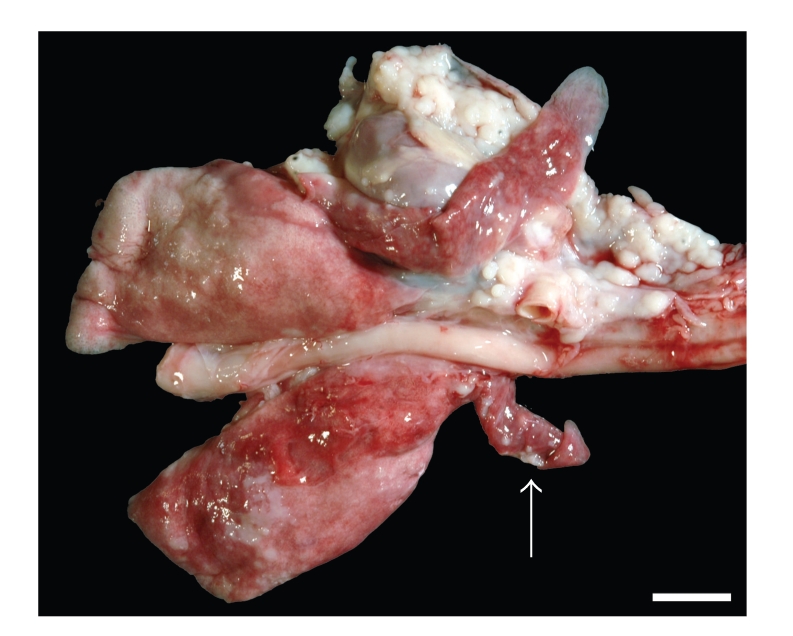
Lung, cranial mediastinum, and pericardium. Serosal surfaces are covered by whitish nodules. The same nodules are discernible within the right cranial lung lobe (arrow). Additionally the cranio-ventral parts of the lung show atelectasis, while dorsal parts of the lung show alveolar emphysema. Bar *≈* 1 cm.

Necropsy of the minimally anaemic animal revealed 50 mL of a clear serosanguineous liquid within the thoracic cavity. The surface of the thoracic cavity including the diaphragm and cranial mediastinum was covered multifocally to coalescing by firm whitish smooth nodules of up to 6 × 5 × 3 mm in size (Figure 1). These nodules were especially prominent on the sternal surface of the pleura especially within and around the sternal lymph nodes. On cut section the nodules appeared homogenously whitish and were mostly firmly attached to the underlying tissue. The right cranial lung lobe contained identical nodules. Additionally, the cranial and the ventral parts of the caudal lung lobes were atelectatic. The dorsal aspects of the caudal lobes comprised a moderate, multifocal, acute, alveolar emphysema (Figure 2).

In addition, a nodule of 7 × 7 × 6 mm was discovered within the right caudal lung lobe. 

Additional findings included a moderate left ventricular, myocardial hypertrophy, a mild, multifocal, chronic, suppurative pericholangitis and moderate, multifocal,and nodular, hyperplasia of the pancreas. 

**Figure 3:**
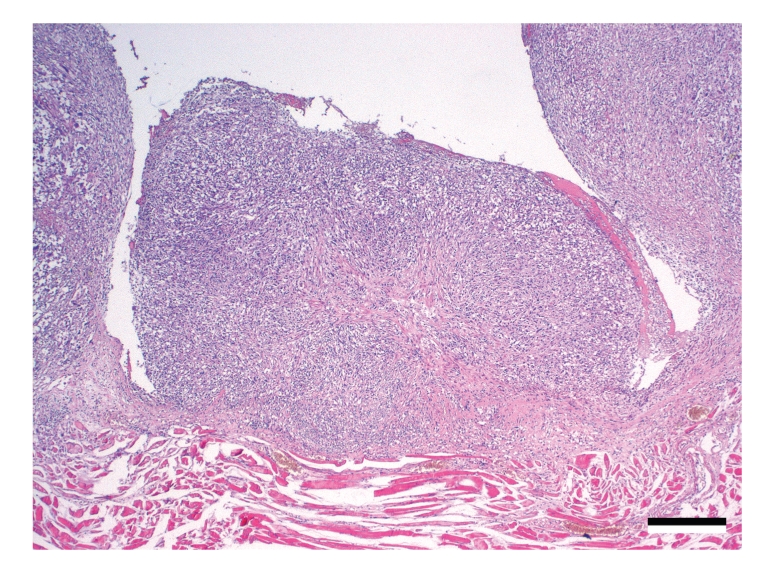
Thoracic wall (H.E.). A nodule composed of spindle-shaped cells arranged in streams and sheets displaying invasion into the underlying muscle in the lower right corner is shown. Bar *≈*400 *μ*m.

**Figure 4:**
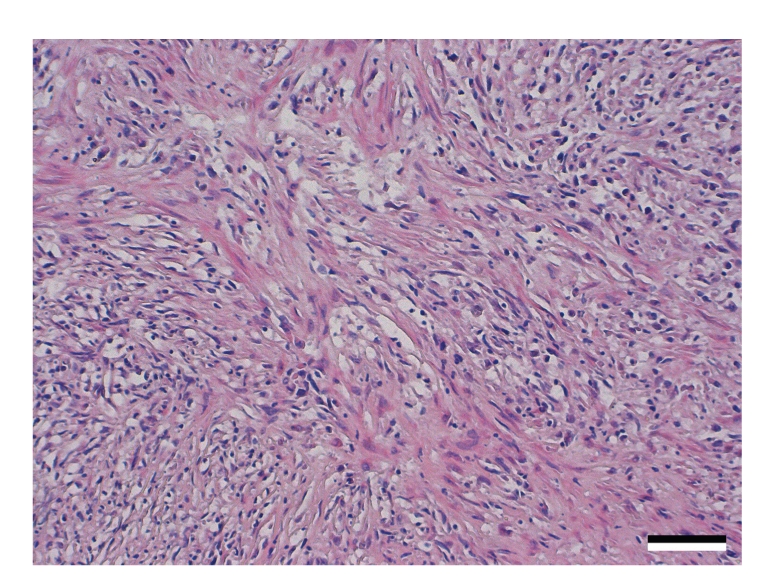
Higher magnification of the fibrosarcoma-like neoplasm. Bar *≈*80 *μ*m.

Histopathological examination of haematoxylin and eosin-stained sections of the thoracic wall revealed several invasively growing, unencapsulated masses of neoplastic cells (Figure 3). Tumour cells were arranged in sheets and streams supported by a prominent fibro-vascular stroma. Neoplastic cells were predominantly spindle-shaped with moderate amounts of eosinophilic cytoplasm and ovoid nuclei. Polygonal cells contained round nuclei (Figure 4). Nuclei were mildly hyperchromatic and contained finely stippled chromatin and no discernible nucleoli. Multifocally binucleated and multinucleated neoplastic cells were present. The cranial sternal lymph node was replaced by the same tissue. The right cranial lung lobe contained identical nodules. 

Additional the bigger nodule in the right caudal lung lobe was identified as pulmonary adenocarcinoma of the acinar type. In this nodule moderately well-differentiated epithelial cell formed palisades and acinar structures on a moderately prominent fibro vascular stroma. 

**Figure 5:**
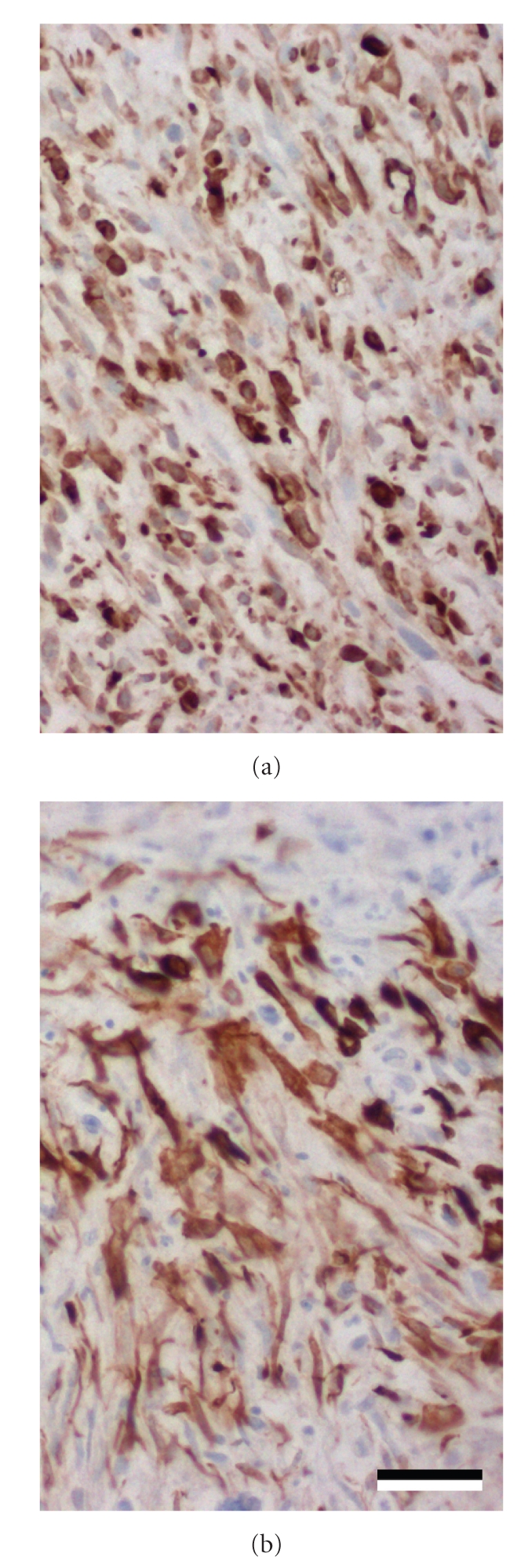
Immunohistochemistry for mesenchymal ((a) vimentin) and epithelial ((b) pancytokeratin) markers is positive indicating a mesothelioma. Bar *≈* 40 *μ*m.

Fibrosarcoma was the preliminary diagnosis of the tumour according to its histologic features. To ascertain the diagnosis immunohistochemistry for cytokeratin (pancytokeratin antibody, clone AE1/AE3, DAKO, Hamburg, Germany) and vimentin (clone Vim 3B4, DAKO, Hamburg, Germany) by standard methods was conducted. Neoplastic cells expressed both the epithelial marker cytokeratin and mesenchymal marker vimentin (Figure 5), except some, multinucleated cells that had only weak cytokeratin expression. Interestingly, spindle cells displayed more prominent cytokeratin expression than the polygonal cells. However, both cell types had equally prominent expression of vimentin. 

Due to the immunohistochemical features of the tumour the final diagnosis of a predominantly fibrous malignant mesothelioma was made.

In contrast, pulmonary adenocarcinoma cells exclusively expressed cytokeratin, wheras stromal cells only expressed vimentin.

## 3. Discussion

Mesotheliomas are rare predominantly malignant neoplasms of aged domestic cats [1]. As is the case in man, it can be quite challenging to distinguish epithelioid mesotheliomas from metastatic carcinomas, fibrous malignant mesotheliomas, fibrosarcomas or synovial cell sarcoma by histological examination alone [1, 4, 5]. However, diagnosis can be supported by unique immunohistological features of mesotheliomas. A constant feature of these neoplasms is the coexpression of epithelial and mesenchymal markers, like cytokeratin and vimetin [1]. In ambiguous cases as this one, this can help to reach the correct diagnosis even of fine needle aspirates. However, it has to be kept in mind, that de-differentiated pulmonary adenocarcinomas, also potentially express vimentin [1], but this was not the case in the tumour described here. Electron microscopy may be a further method to support the diagnosis by demonstrating typical mesothelial type microvilli [1]. Gross appearance in our case was typical for mesothelioma, but histological appearance was misleading. This underlines the significance of immunohistological differentiation of certain neoplasms.

The majority of the reported feline mesotheliomas affected the thoracic cavity [1, 2, 6] similar to the case presented here. Additionally most cases were associated with serous to serosanguineous effusions and metastasis to adjacent organs. In this case signs of malignancy were invasive behavior and particularly metastasis to the sternal lymph node and the lung. Common clinical symptoms in affected animals are therefore dyspnoea, weight loss, and coughing. 

Diagnosis by cytologic features alone may be difficult, as cytological differentiation of activated mesothelial cell and neoplastic mesothelial cells is very difficult. For instance activated mesothelial cells may become pleomorphic and may form multinucleated giant cells [7].

Aetiology of spontaneous mesothliomas in domestic animals is unknown. In man asbestos exposure is closely associated with the development of mesotheliomas [5]. However in domestic animals the relation between asbestos exposure and development of mesotheliomas could not be proven thus far [1], but experimentally asbestos exposure of laboratory animals also leads to the development of mesotheliomas [8]. 

Even in human beings there is no effective treatment for mesotheliomas until now [4]. Surgical excision of mesotheliomas is the only available treatment option. Nevertheless, at late stages of tumour development the success rate of surgery is low due to the highly infiltrative growth of this tumour type and the abundant contact metastases.

Prognosis in animals with malignant mesothelioma is guarded to unfavourable. Possible palliative therapeutic measures include pericardio- or thoracocenthesis as well as intracavital instillation of carboplatin [9]. Small and focal lesions can be surgically excised. Chemotherapy using doxorubicin or intracavital application of cisplatin is reported to be helpful [10]. 

In conclusion mesothelioma has to be kept in mind as differential diagnosis in cats with dyspnoea, thoracic effusions, and thickening of the pleura. Because it is a rare neoplasm and different subtypes can be misinterpreted as carcinomas or sarcomas, respectively, immunohistochemistry should be included in the diagnostic procedures.
